# Cefazolin sodium pentahydrate cause urolithiasis: a case report and literature review

**DOI:** 10.1093/jscr/rjac115

**Published:** 2022-04-11

**Authors:** Fujun Wang, Wen Nie, Zongjun Wang, Sujian Tian, Junqiang Dong

**Affiliations:** Department of Urology, Heze Municipal Hospital, Shandong Province, Heze, China; Department of Surgery, Jiaozhou Hospital of Tongji University Dongfang Hospital, Shandong Province, Qingdao, China; Department of Testing Inspection, Heze Medical College, Shandong Province, Heze, China; Department of Urology, Heze Municipal Hospital, Shandong Province, Heze, China; Department of Urology, Heze Municipal Hospital, Shandong Province, Heze, China

## Abstract

We report a rare case of urolithiasis induced by cefazolin sodium pentahydrate and review the relevant literature. A 12-year-old girl with right kidney injury was admitted to our hospital, a computed tomography scan revealed that no signs of disease in her left kidney but her right kidney was traumatized severely. After receiving cefazolin sodium pentahydrate, 2.0 g by intravenous infusion daily for 10 days, urolithiasis was found in the left urinary tract by computed tomography scan. Later, the patient complained of left back pain, nausea and vomiting, and a further computed tomography scan showed calculi persisted in the left urinary tract, and some of which had caused left hydronephrosis. A double-J catheter was placed in the left ureter, but no calculi were seen to drain with urine in the next 2 weeks, those calculi were removed by a flexible ureteroscope.

## INTRODUCTION

Cephalosporins are beta-lactam-class antibiotics widely used in clinical practice because of their strong antibacterial activity. There are currently five generations of cephalosporins, mainly differentiated according to their structure, spectrum of activity and side-effect profile [1]. Cefazolin sodium pentahydrate, which belongs to the first generation of cephalosporin antibiotics, is an upgraded form of the traditional cefazolin sodium that further extends the strong anti-Gram-positive bacteria action of cefazolin sodium [2] and is suitable for the treatment of urinary tract, respiratory tract and biliary system infections. Previous studies have shown that cephalosporins such as ceftriaxone, cefotaxime and ceftazidime could induce urolithiasis [3–5]. Upon reviewing the available literature, we found no reports of urolithiasis induced by cefazolin sodium pentahydrate. We report the case of a young girl with right kidney injury who developed multiple calculi in the left kidney after treatment with cefazolin sodium pentahydrate.

## CASE REPORT

A 12-year-old girl with right kidney injury was admitted to our hospital on 30 January 2021. A computed tomography (CT) scan before admission showed that no abnormalities in her left kidney but her right kidney was traumatized severely. The laboratory tests revealed that white blood cells were 14.86 × 10^9^/L, red blood cells were 2.33 × 10^9^/L, serum calcium was 1.86 mmol/L and kidney function was normal.

**Figure 1:**
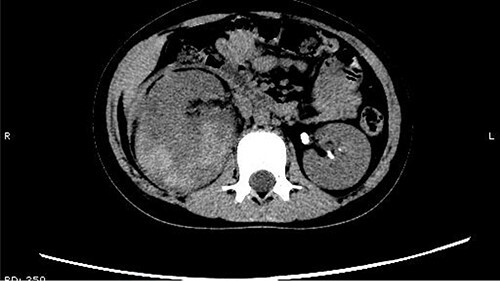
Some irregular high-density shadows was present in the left renal pelvis.

**Figure 2:**
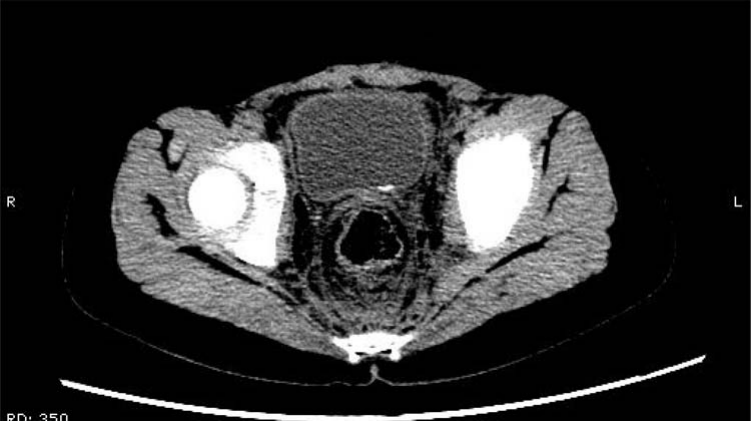
A small strip of high-density shadow was present in the bladder cavity.

The patient was confined to bed, fasted and prescribed a daily intravenous infusion of cefazolin sodium pentahydrate, 2.0 g for 10 days. CT showed some irregular high-density shadows were present in the left renal pelvis (Fig. 1), and a small strip of high-density shadow was apparent in the bladder cavity (Fig. 2). Blood tests showed no abnormality in serum calcium concentration. Cefazolin sodium pentahydrate was discontinued immediately.

**Figure 3:**
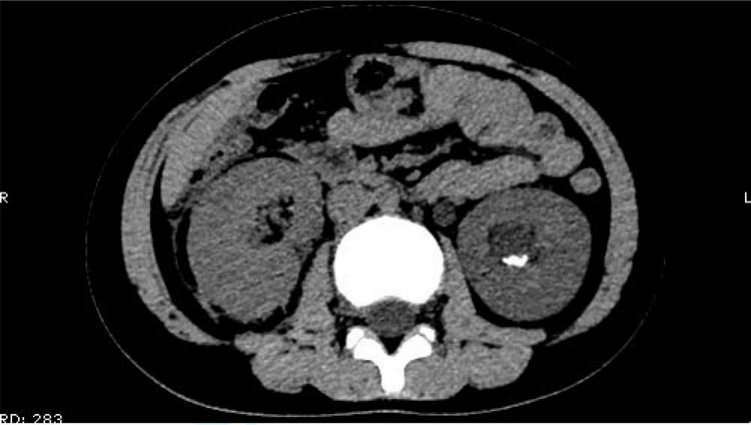
Some irregular high-density shadows persisted in the left renal pelvis, hydronephrosis was apparent in the left renal pelvis.

**Figure 4:**
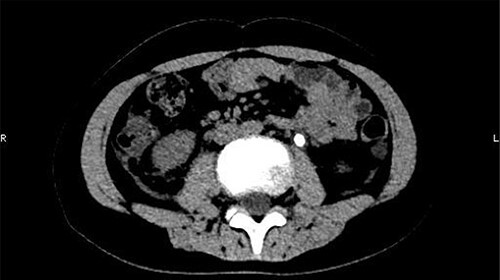
An irregular high-density shadow was present in the left upper ureter.

On 25 February, the patient complained of left back pain, nausea and vomiting. A further CT scan showed some irregular high-density shadows persisted in the left renal pelvis (Fig. 3) and an irregular high-density shadow was present in the left upper ureter (Fig. 4); furthermore, hydronephrosis was apparent in the left renal pelvis. There were no abnormalities in the bladder.

**Figure 5:**
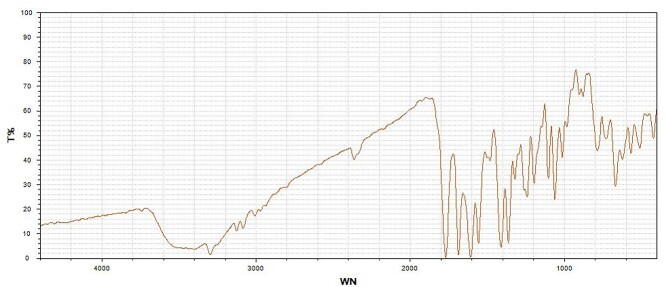
The calculi were confirmed as cephalosporin-induced nephrolithiasis by infrared spectrum analysis. T, transmittance; WN, wavenumber.

A double-J catheter was placed immediately in the left ureter, after which the symptoms of renal colic disappeared. No calculi were seen to drain with the urine over the next 2 weeks, then it was decided to remove the calculi by a flexible ureteroscope. These calculi resembled glue and were confirmed as cephalosporin-induced nephrolithiasis by infrared spectrum analysis (Fig. 5). No recurrence of urolithiasis was observed over the follow-up period of 6 months.

## DISCUSSION

Drug-induced urolithiasis represents 1–2% of all renal calculi [6, 7], for which a variety of commonly used drugs are responsible. Use of antibiotics is a contributing factor in the development of urolithiasis. Sulfa drugs, cephalosporins, fluoroquinolones, nitrofurantoin/methenamine and broad-spectrum penicillins prospectively increase the risk of urolithiasis [8, 9]. In the present case, urolithiasis was rarely induced by cefazolin sodium pentahydrate.

Cephalosporins can cause renal injury, which can facilitate the formation of urinary calculi [3]. Chatchen *et al*. [10] studied the underlying pathophysiology of ceftriaxone-associated acute kidney injury via cellular proteomic *in vitro* cell model approach, whereby cell survival and adaptation were mediated by the concurrent upregulation of Hsp70, whereas downregulation of annexin A1 was proposed as the negative-feedback mechanism to mitigate the stressful effects of ceftriaxone crystallization, cell growth arrest and delayed wound healing of distal tubular cells after ceftriaxone crystal exposure, shedding light on collateral damage in combination therapy involving ceftriaxone and other stressors. Kimata *et al*. [11] demonstrated that ceftriaxone had the potential to significantly increase urinary excretion of calcium, which might be linked to ceftriaxone-related urolithiasis or sludge. We deduce that cefazolin sodium pentahydrate may have a similar mechanism of calculi formation induced by ceftriaxone.

Cefazolin sodium pentahydrate is scarcely metabolized in the body, the prototypical drug being completely eliminated by the kidneys. In the present case, the patient’s right kidney was traumatized severely and cefazolin sodium pentahydrate was almost all discharged through the healthy left kidney. The higher concentration of drug in the healthy kidney may have caused cell injury in the left kidney and facilitated the formation of calculi. Dehydration can lead to decreased circulation volume and oliguria, which poses a risk for calculi formation [3]. Murata *et al*. found that fasting and bed rest, even for a relatively short period, were risk factors for ceftriaxone-associated pseudolithiasis [12]. In our case, the patient was dehydrated because of severe trauma and had to be confined to bed and fasted for the same reason, which may also have contributed to the formation of urolithiasis. Most reported patients with ceftriaxone-induced urolithiasis are children, probably because a child’s urinary system is immature and cannot yet function optimally [3]. The current patient was 12 years old, consistent with the existing literature.

The diagnosis of drug-induced urolithiasis mainly relies on drug-intake history, clinical symptoms and imaging examination. When a patient presents with symptoms of renal colic such as back pain, nausea and vomiting with a suspicion of drug-induced urolithiasis, medical history should be the first line of inquiry, followed by a CT or ultrasound examination. CT scan is superior to ultrasonography because it can better show the place, size and hardness of calculi.

Given that ceftriaxone-related urolithiasis are small and sand-like with a loose texture, generally after discontinuation of the drug, the calculi can be drained with the urine spontaneously [6, 13]. However, during the process of calculi passing through the ureter, if the calculi block the ureter to cause back pain or anuria, a ureteral stent should be placed in the blocked ureter [14, 15]. However, for those patients who still have residual calculi after these measures are taken, ureteroscopy should be performed.

In conclusion, cephalosporin antibiotics carry a potential risk of inducing urolithiasis, cefazolin sodium pentahydrate should be used with caution in patients with renal trauma or a single kidney to avoid the occurrence of such an adverse event. We hope that this case report and literature review will arouse the attention of clinicians.
